# S22. A TRANSDIAGNOSTIC NEUROANATOMICAL SIGNATURE OF PSYCHIATRIC ILLNESS

**DOI:** 10.1093/schbul/sby018.809

**Published:** 2018-04-01

**Authors:** Gong Qiyong, Cristina Scarpazza, Jing Dai, Manxi He, Xin Xu, Yan Shi, Baiwan Zhou, Sandra Vreira, Eamon McCrory, Cheng Yang, Feifei Zang, Su Lui, Andrea Mechelli

**Affiliations:** 1 Huaxi MR Research Center (HMRRC), West China Hospital of Sichuan University; 2 Institute of Psychiatry, Psychology and Neuroscience, King’s College London; 3 Chengdu Mental Health Center; 4 University College London

## Abstract

**Background:**

Despite an increasing research and clinical focus on trans-diagnostic approaches to mental health, it remains unclear whether different diagnostic categories share a common neuronatomical basis. The current investigation sought to investigate whether a shared (trans-diagnostic) set of structural alterations characterized schizophrenia, depression, post-traumatic stress disorder and obsessive-compulsive disorder, and determine whether any such alterations reflected markers of psychiatric illness or pre-existing familial vulnerability.

**Methods:**

A total of 404 patients with a psychiatric diagnosis were recruited (psychosis, n=129; unipolar depression, n=92; post-traumatic stress disorder, n=91; obsessive compulsive disorder, n=92) alongside 201 healthy controls and 20 unaffected first-degree relatives. We collected structural Magnetic Resonance Imaging scans from each participant using the same 3.0 Tesla scanner and acquisition sequence, and tested for trans-diagnostic alterations using Voxel-Based Morphometry.

**Results:**

We report that the four psychiatric groups were all characterized by significantly greater gray matter volume in the bilateral putamen relative to healthy controls (right putamen: x=32 y=6 z=-2; z-score: 5.97; p-value<0.001 after FWE-correction; left putamen: x=-30 y=5 z=-7; z-score: 4.97; p-value=0.001 after FWE-correction). Furthermore, the volume of this region was positively correlated with severity of symptoms across groups, so that a higher gray matter volume in the putamen was associated with higher severity of symptoms (partial correlation: the results are age and gender corrected; right putamen: r=0.313, p<0.001; left putamen: =0.326, p<0.001). Bilateral putamen enlargement was also evident in unaffected relatives compared to healthy controls (right putamen: x=32 y=-6 z=2; t-value: 8.13, p-value<0.001 after FWE-correction; left putamen: x=-32 y=-8 z=2; z-score:9.39, p-value<0.001 after FWE-correction).

**Discussion:**

These findings indicate that increased putamen volume may reflect a trans-diagnostic marker of underlying familial vulnerability to psychopathology. This raises the prospect that this region could be used to assess degree of familiar vulnerability to psychopathology above and beyond traditional diagnostic boundaries. Our investigation provides support to emerging conceptualisations of psychiatric illness, in which each disorder is best understood as a combination of diagnosis-specific features and a trans-diagnostic factor reflecting general psychopathology.

